# A prospective study to validate the functional assessment of cancer therapy (FACT) for epidermal growth factor receptor inhibitor (EGFRI)-induced dermatologic toxicities FACT-EGFRI 18 questionnaire: SWOG S1013

**DOI:** 10.1186/s41687-020-00220-x

**Published:** 2020-07-08

**Authors:** Siu-Fun Wong, Joseph M. Unger, James L. Wade, Lynne I. Wagner, Mario E. Lacouture, Keisha C. Humphries, Anna Moseley, Kathryn Arnold, Mario R. Velasco, Justin D. Floyd, Benjamin T. Esparaz, Afsaneh Barzi, Heinz-Josef Lenz, Marianna Koczywas, Shaker Dakhil, Gary V. Burton, Michael J. Fisch, N. Lynn Henry, Dawn L. Hershman, Carol M. Moinpour

**Affiliations:** 1 Chapman University School of Pharmacy, Rinker Health Science Campus, 9401 Jeronimo Road, Irvine, CA USA; 2 SWOG Statistics and Data Management Center/Fred Hutchinson Cancer Research Center, Seattle, WA USA; 3 Heartland Cancer Research NCORP/Cancer Care Specialists of Central Illinois, Decatur, IL USA; 4 Wake Forest School of Medicine, Winston Salem, NC USA; 5 Memorial Sloan-Kettering Cancer Center, New York, NY USA; 6 Wichita NCORP, Wichita, KS USA; 7 Heartland Cancer Research NCORP/Cancer Care Specialists of Central Illinois, Swansea, IL USA; 8 University of Southern California, Los Angeles, CA USA; 9 City of Hope, Duarte, CA USA; 10 Wichita NCORP/ Cancer Center of Kansas, Wichita, Wichita, KS USA; 11 Gulf South MU NCORP/Louisiana State University HSC-Shreveport, Shreveport, LA USA; 12 Aim Specialty Health, Chicago, IL USA; 13 University of Michigan Medical School, Ann Arbor, MI USA; 14 Columbia University Medical Center, New York, NY USA; 15 Fred Hutchinson Cancer Research Center, Public Health Sciences Division (Emerita), Seattle, WA USA

**Keywords:** EGFRI, FACT-EGFRI 18, Dermatologic toxicity, Papulopustular rash, Patient-reported outcome measure, Health-related quality of life, HRQL

## Abstract

**Background:**

Papulopustular rash is a common class effect of epidermal growth factor receptor inhibitors (EGFRI) that can affect patients’ health-related quality of life and cause disruptions to treatment. SWOG S1013 (NCT01416688) is a multi-center study designed to validate the Functional Assessment of Cancer Therapy EGFRI 18 (FACT-EGFRI 18) using 7-items from the National Cancer Institute (NCI) Common Terminology Criteria for Adverse Events (CTCAE) version 4.0 to assess EGFRI-induced skin-related toxicities and their impact on functional status.

**Methods:**

Patients with a diagnosis of colorectal or lung cancer to receive EGFRI therapies for at least 6 weeks were enrolled. Patient self-assessments using the FACT-EGFRI 18 were completed prior to undergoing CTCAE assessment by trained clinicians at baseline, weekly × 6, and then monthly × 3. The psychometric properties of the FACT-EGFRI 14 (skin toxicity items only) and 18 (plus 2 nail and 2 hair items) were established based on criterion validity, known groups validity, internal consistency reliability, and responsiveness to change.

**Results:**

Of the 146 registered patients, 124 were evaluable. High Cronbach’s alpha (> 0.70) for both FACT-EGFRI 14 and FACT-EGFRI 18 scores across assessment times were observed. Although agreement (i.e. criterion validity) between individual and summary scales of the FACT-EGFRI 18 for assessing skin toxicity was good, agreement with the clinician-reported CTCAE was only fair. The minimal important difference was determined to be 3 points. The results also demonstrated responsiveness to symptom change.

**Discussion:**

Based on the results of this multi-center validation study, the FACT-EGFRI 18 patient-reported outcome instrument provided data from the patient’s perspective yielding unique information as well as complementing clinician-rated CTCAE grades, especially for the symptoms of pain, pruritus, and paronychia.

**Conclusions:**

Good to excellent psychometric properties for the FACT-EGFRI 18 were demonstrated, supporting further use of this patient-reported outcomes measure. Additional validation with a more diverse group of patients should be conducted.

## Background

A characteristic rash has been documented to be a “class adverse effect” of agents that target the epidermal growth factor receptor (EGFR), including the monoclonal antibodies (Mab) cetuximab and panitumumab, and the tyrosine kinase small molecule inhibitor (TKI) erlotinib [1]. Approximately 90% and 75% of cancer patients receiving EGFR inhibitors (EGFRI) Mabs and TKIs, respectively, develop a papulopustular eruption within the first 2–3 weeks after the start of therapy, and the toxicity is often dose-dependent [2–4]. The eruption is characterized by inflammatory papules and pustules most often seen on the face, chest, and back but occasionally extending to the extremities; scratching due to pruritus can cause secondary infection. These lesions may resemble folliculitis or an acneiform drug eruption with tenderness and pruritus. Multiple large phase III trials of EGFRI therapies have found drug-related papulopustular rash in 75% of patients, including 8% with grades 3–4 toxicity [5, 6]. The discomfort and physical appearance of this common toxicity affect patients’ instrumental activities of daily living (IADL) and health-related quality of life (HRQL), and can cause disruptions to treatment [7–9].

Interest in rash management has increased over time due to published data suggesting a possible relationship between the presence of rash and treatment response and/or patient survival [8, 10–13]. The current management approach is either prophylactic with oral antibiotics and topical corticosteroids or reactive treatment with dose modifications or discontinuation of EGFRIs upon the occurrence of intolerable Grade 2 or Grade 3 or 4 skin toxicity. No standardized treatment regimen has been identified as the optimal approach to prevent or treat EGFRI-induced skin toxicity. Research to advance knowledge and establish consensus to optimally manage EGFRI-induced skin rash requires universally accepted, reliable, and validated patient reported outcome (PRO) measures to complement the National Cancer Institute Common Toxicity Criteria and Adverse Events (CTCAE) [14–16]. measures add the impact of skin toxicities and functional status assessments.

Between 2010 to 2017, three other grading systems were proposed [17–19]. None of these systems has been adopted as the standard of care tool by the medical community. The National Cancer Institute’s PRO-CTCAE was an important addition to the small set of patient-reported symptom measures addressing skin toxicity symptoms, but were not available when S1013 was developed [20–24]. One concern is these items do not thoroughly address the full range of skin toxicities on functional impacts. The patient-reported instrument used in this trial, the Functional Assessment of Cancer Therapy EGFRI 18 (FACT-EGFRI 18) as shown in Table 1 [25], does address these broad symptoms, but had not been fully validated. Therefore, there remained a need to validate a PRO measure to capture patients’ experiences with treatment and effects on HRQL, enhance clinicians’ ability to accurately assess the severity and effect of skin toxicity symptoms, and evaluate interventions to prevent or manage skin toxicities.

**Table 1:** The Functional Assessment of Cancer Therapy-EGFRI 18 (FACT-EGFRI 18)

Instructions: “Below is a list of statements that other people with your illness have said are important. Please check one box per line to indicate your response as it applies to the past 7 days.”						
Item No.	Item	Response category				
		Not at all	A little bit	Somewhat	Quite a bit	Very much
1	My skin or scalp feels irritated					
2	My skin or scalp is dry or “flaky”					
3	My skin or scalp itches					
4	My skin bleeds easily					
5	I am bothered by a change in my skin’s sensitivity to the sun					
6	My skin condition interferes with my ability to sleep					
7	My skin condition affects my mood					
8	My skin condition interferes with my social life					
9	I am embarrassed by my skin condition					
10	I avoid going out in public because of how my skin looks					
11	I feel unattractive because of how my skin looks					
12	Changes in my skin condition make daily life Difficult					
13	The skin side effects from treatment have interfered with household tasks					
14	My eyes are dry					
15	I am bothered by sensitivity around my fingernails or toenails					
16	Sensitivity around my fingernails makes it difficult to perform household tasks					
17	I am bothered by hair loss					
18	I am bothered by increased facial hair					

Note: FACT-EGFRI 18 measure and scoring instructions are available at the following website: https://www.facit.org/FACITOrg/Questionnaires

Because of the absence of a validated or “gold standard” PRO measure to assess EGFRI skin toxicity, a multi-center study was designed to evaluate the psychometric properties of the FACT-EGFRI 18 [25]. The goal was to obtain a more comprehensive assessment of EGFRI-induced skin-related toxicities, particularly with respect to impact on patient functional status, in order to inform and promote optimal supportive care as well as continued research for this patient population.

## Methods

### Study design

#### Patients

The trial was conducted by the SWOG (formerly the Southwest Oncology Group) Cancer Research Network, a National Cancer Institute (NCI)-sponsored National Clinical Trials Network group and a member of the NCI’s Community Oncology Research Program. Eligible patients, screened and recruited by investigators from 11 SWOG sites, had a diagnosis of colorectal or lung cancer and were planning to receive one of the following EGFRI treatment regimens for at least 6 weeks: 1) Cetuximab 400 mg/m^2^ IV loading dose, 250 mg/m^2^ IV weekly; 2) Cetuximab 500 mg/m^2^ IV every 2 weeks; 3) Panitumumab 6 mg/kg IV every 2 weeks; or 4) Erlotinib 100–150 mg PO daily. Concurrent chemotherapy was allowed, except for agents known to cause rash that could interfere with EGFRI-induced skin toxicity assessment. Patients receiving prior EGFRI therapy must have fully recovered from any skin toxicities prior to registration. Patients must not have been planning to receive concomitant medications or treatments that could cause rash or other dermatologic reactions (including radiation therapy) and must not have any serious concomitant skin disorder (such as eczema) that could interfere with assessment of EGFRI-induced skin toxicity. Patients had a Zubrod [26] performance status of 0–2. Patients completed questionnaires in English.

All patients were informed of the investigational nature of this study and were required to sign and give written informed consent in accordance with institutional and federal guidelines.

#### Patient-completed measures: FACT-EGFRI 18 questionnaire

The FACT-EGFRI 18 [25] is a PRO measure to assess dermatologic-related symptom burden and HRQL among patients receiving EGFRIs. It includes 18 items evaluating the physical, functional, emotional, and social impact of symptoms in three anatomical areas: skin with 14 items, which comprises the FACT-EGFRI 14, plus 2 nail items and 2 hair items. In accordance with standard FACT administration procedures [27], patients rate each item using a 5-point Likert scale from 0 (Not at all) to 4 (Very much) based on the past 7 days. Based on the Functional Assessment of Chronic Illness Therapy (FACIT) scoring system, a higher score indicates better HRQL (e.g. lower severity of symptoms) [25]. Patient assessments were completed prior to physician assessments at each visit. The assessment timepoints occurred prior to EGFRI initiation at baseline (Day 1) and on Days 8, 15, 22, 29, 36, and 43; and then monthly for 3 months on Days 71, 99 and 127. Assessments continued through Day 127 even if EGFRI treatment was delayed or discontinued.

#### Patient-completed measures: anchor items

This two-item patient completed measure addresess the patient’s sense of the degree of change in the severity of skin symptoms and the degree of change in the impact of skin symptoms on the patient’s daily life since the last time questionnaires were completed [28]. These two items were used to help estimate a mean level of change in symptom severity and impact experienced by patients in S1013. The severity item response for minimal level of change was a little bit better or a little bit worse (representing a one-point change in either direction). Selection of zero reflected the patient’s sense that symptom severity was about the same. Very much change (very much better or worse) was represented by selection of + 2 or − 2. Instead of the mean minimum difference, the overall mean difference is reported. The same definitions held for the change in the impact of skin symptoms on daily life. Patients did not complete the anchor items at the prestudy assessment time but began completing this form with the second assessment at day 8. The mean score for each anchor choice through day 43 for severity and impact was calculated for a single mean score for change in severity and change in impact. The results reported in this study intend to emphasize the mean overall change in skin toxicity symptom severity and skin toxicity symptom impact and not the mean minimum difference.

#### Clinician-completed measures: CTCAE items

Seven National Cancer Institute Common Toxicity Criteria and Adverse Events (CTCAE) items [15] (Rash acneiform, Pruritus, Dry skin, Pain of skin, Paronychia, Alopecia, and Hypertrichosis) addressed criterion validity for the FACT-EGFRI-18 (Online Appendix Table 1). These seven items are most commonly associated with skin toxicity assessment. The grading of the CTCAE ranges from Grades 1 through 5 with unique clinical descriptions of severity for each adverse event defined as: Grade 1 = Mild, Grade 2 = Moderate, Grade 3 = severe, Grade 4 = Life-threatening or disabling, and Grade 5 = Death. Although the NCI CTCAE items do not have psychometric documentation similar to that provided for patient-reported measures, they are widely accepted as physician/clinician measures of adverse events in cancer clinical trials. To minimize variability in the ratings, only physician investigators and the nurse/clinical research associate at each site who had previously undergone formal training in the CTCAE assessments by SWOG rated the subjects’ EGFRI-induced skin toxicities.

#### Clinician-completed measure: performance status items

The Zubrod Scale is the performance status (PS) measure developed by CG Zubrod for use in cancer clinical trials [26]. It is the measure used in the Eastern Cooperative Oncology Group (ECOG) - American College of Radiology Imaging Network (ACRIN) trials [29]. This PS measure is a 5-point scale from normal status to unable to get out of bed. In S1013, the Zubrod provided a “history” of PS over the course of the study.

### Statistical considerations

The overall goal of this study was to evaluate the psychometric properties of the FACT-EGFRI 18 [25] based on criterion validity, known groups validity, internal consistency reliability, and responsiveness to change. To be considered evaluable for endpoint analyses, patients must have met all eligibility criteria, completed the baseline FACT-EGFRI 18 prior to the start of EGFRI treatment, and had valid anchor assessments (Days 1, 8, 15, or 22) and valid follow-up assessments (Days 29, 36, or 43). A valid assessment is one where the FACT-EGFRI 18 is completed prior to the physician’s evaluation of skin toxicity. The analysis focused on the psychometric properties of the FACT-EGFRI 18 [25] through the Day 43 assessment, corresponding to the minimum required treatment time for eligibility.

#### Criterion validity

Because there is no validated or “gold standard” PRO measure available in this setting, criterion validity was defined as agreement between the FACT-EGFRI 18 [25] and the CTCAE [15] scoring system. Agreement was measured using an unweighted Kappa statistic, with moderate or better agreement defined as Kappa [30] coefficients of ≥0.41. Agreement was assessed between the seven treatment toxicity categories most commonly associated with EGFRI toxicity and the total and individual scores from the FACT-EGFRI 18 items. To enable comparison between the EGFRI score and the CTCAE grade using a Kappa statistic [30], total scores for the EGFRI categories were generated on a scale from 0 to 100. These scores were then categorized into the number of respective CTCAE levels using ranks, with cell sizes defined by the patients’ corresponding CTCAE score distributions. Constructing marginal distributions for EGFRI total scores in this fashion avoids arbitrary categorization (since it relies on the observed distribution of CTCAE scores) and also allows for the contingency of perfect concordance (a desirable property, which is highly unlikely). Due to the fact that patients could enter this study with none to any level of skin toxicity and that approximately 80% of patients receiving the EGFRI agents develop skin toxicity, we expected to observe a broad range of such toxicities including none. Only the sixteen items that explicitly corresponded to CTCAE items were used for the criterion validity analyses. A table showing items from both measures is included in the online appendix.

#### Known groups validity

Known groups validity [31–33] was assessed by examining differences in mean EGFRI subscores (eg. rash, pruritus, pain) between patients who reported a PS of 0 (indicating normal activity without symptoms) and patients who reported feeling any symptoms (PS > 0). ANOVA was used to compare FACT-EGFRI 18 scores between groups, with differences of 1/3 to 1/2 of a standard deviation considered of interest [31]. The a priori hypothesis was that mean scores of the FACT-EGFRI 18 skin adverse events measure would be significantly higher (better) for lower (better) performance status (based on reported symptom level).

#### Internal consistency reliability

Internal consistency reliability was evaluated using Cronbach’s alpha [34]. Coefficients of > 0.70 are considered sufficient evidence of reliability [35]. Reliability was assessed for two scores: the FACT-EGFRI 14 score for the 14 skin toxicities and the full FACT-EGFRI 18 scale that included two nail and two hair toxicity items in addition to the 14 skin toxicities.

#### Responsiveness to change

Longitudinal regression using linear mixed models (wth patient treated as a random effect) was used to measure change in FACT-EGFRI 18 scores over time, with 1/3 to 1/2 of a standard deviation reflecting a clinically significant change [31].

#### Minimal important difference (MID)

The MID for the FACT-EGFRI 18 scores was estimated by comparing unweighted mean changes in the FACT-EGFRI 18 score to the patient’s direct assessment of change between adjacent levels of two anchor items, severity and impact [28].

Criterion validity, known groups validity and reliability were described at each timepoint and overall. Secondary objectives included the assessment of minimally important change and the association of the FACT-EGFRI 18 scores with treatment profiles.

#### Power and sample size

The primary objective was to establish the validity of the new measure through the collection of psychometric properties. The accrual goal was *N* = 140 eligible patients, among whom 80% (*N* = 112) were expected to be evaluable for psychometric endpoints. For a Kappa [36] of 0.7 and 5 levels and a sample size of 112 evaluable patients, there is 95% confidence that the true agreement was at least 0.60 (“good” agreement), assuming equal levels of agreement and disagreement for each of the category levels. Given similar parameters, for a Kappa of 0.4, there is 95% confidence that the true agreement was at least 0.28 (“fair” agreement).

## Results

**Fig. 1:**
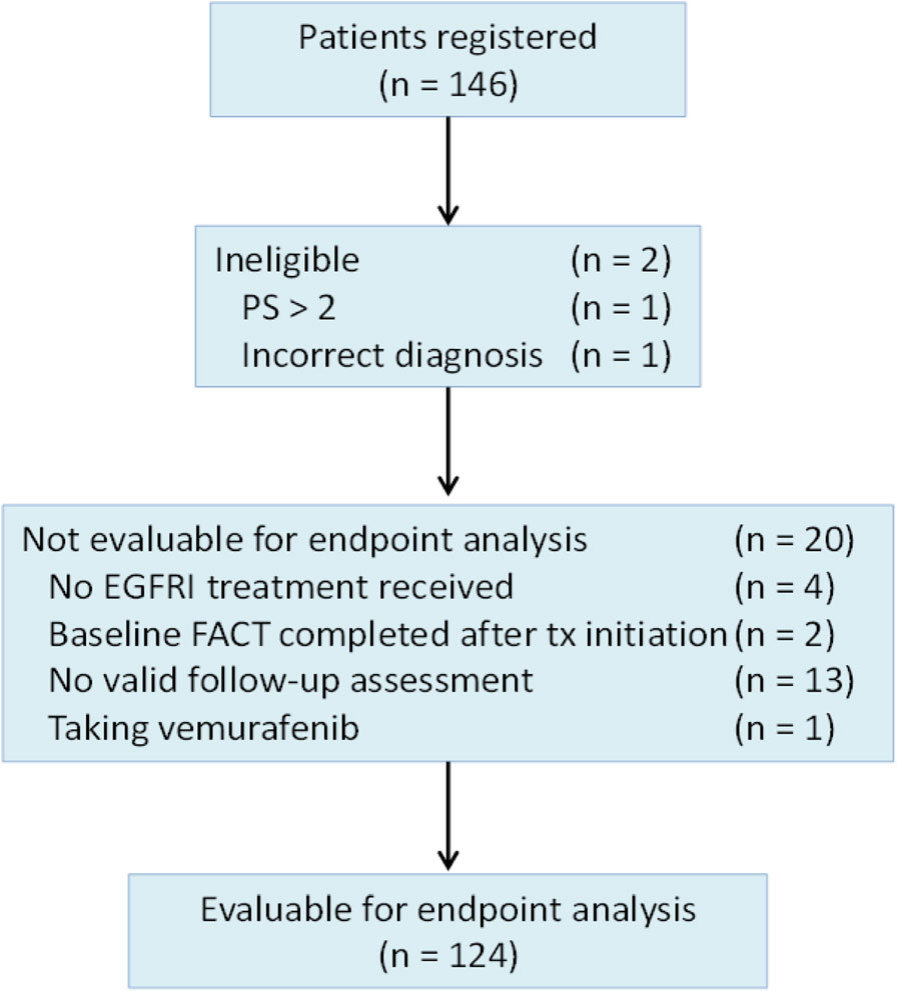
Subject Enrollments

Research staff at 11 institutions enrolled 146 patients between November 15, 2011 and October 1, 2016. Two patients were ineligible: one patient had baseline performance status greater than 2 and the other did not have colorectal or lung cancer. Twenty additional patients were not evaluable (Fig. 1), leaving 124 patients evaluable for the analysis of psychometric endpoints. Evaluable patients were 47% (*n* = 58) male, 16% (*n* = 20) non-white and 10% (*n* = 12) Hispanic, with a median age of 65 years (range 32–88 years). Thirty one patients (25%) had performance status of 0 and 75% (*n* = 93) had performance status > 0. Fifty-four percent (*n* = 67) of evaluable patients were treated with erlotinib, 15% (*n* = 18) with each of the 2 different dose regimens of cetuximab, and 17% (*n* = 21) with panitumumab. Papulopustular rash developed in 112 (90%) of the evaluable patients. Maximum per-patient rash severity ratings based on CTCAE grading were predominantly reported at grade 1 (47%, *n* = 58) and grade 2 (43%, *n* = 53), while 10% (*n* = 12) reported grade 3; the median onset of the rash occurred at 2 weeks. Descriptive statistics for the item and scale scores of the FACT-EGFRI 18 are shown in Table 2.

**Table 2:** Descriptive Statistics for Total FACT-EGFRI 18 Scale and Subscale Scores*

	Mean (SD)									
	Day 1	Day 8	Day 15	Day 22	Day 29	Day 36	Day 43	Day 71	Day 99	Day 127
Alopecia	92.4	93.7	93.5	93.6	91.5	90.1	88.3	82.8	79.9	75.0
	(18.4)	(16.5)	(16.5)	(17.5)	(17.9)	(18.0)	(21.7)	(26.6)	(28.5)	(30.6)
Dry skin	94.4	90.1	82.1	83.6	86.3	88.3	86.6	85.0	84.5	85.6
	(8.7)	(13.9)	(21.7)	(19.3)	(16.9)	(11.5)	(12.6)	(16.1)	(13.4)	(17.4)
Hypertrichosis	96.7	96.1	94.3	95.4	97.4	97.3	95.8	87.8	91.7	86.4
	(11.1)	(13.8)	(17.4)	(11.9)	(8.6)	(8.9)	(12.4)	(23.6)	(22.4)	(24.3)
Pain of skin	95.5	86.6	76.1	79.9	82.4	86.9	82.7	83.0	85.4	84.3
	(11.4)	(18.4)	(23.7)	(22.7)	(20.0)	(12.2)	(16.9)	(18.5)	(16.6)	(18.5)
Paronychia	96.2	95.2	92.5	87.7	86.8	88.0	82.4	77.2	80.2	75.8
	(9.9)	(10.3)	(14.2)	(20.6)	(20.3)	(16.4)	(22.6)	(25.3)	(26.3)	(24.6)
Pruritus	92.8	83.0	74	76.5	78.8	84.9	79.1	80.6	79.2	81.4
	(15.1)	(22.4)	(24.2)	(23.4)	(21.4)	(12.8)	(18.3)	(18.6)	(19.4)	(20.5)
Rash	94.9	89.5	80.7	82.8	85.5	88.5	85.2	84.4	84.3	84.7
	(8.2)	(13.9)	(20.7)	(18.9)	(15.8)	(9.7)	(13.1)	(16.6)	(14.5)	(16.9)
Total FACT- EGFRI 18	67.9	64.9	60.1	60.8	62.1	63.8	61.5	60.1	60.0	59.6
	(5.3)	(8.1)	(11.8)	(10.8)	(9.0)	(6.0)	(8.6)	(11.0)	(9.9)	(11.6)

*Higher FACT-EGFRI scores reflect better health-related quality of life

### Criterion validity

**Fig. 2:**
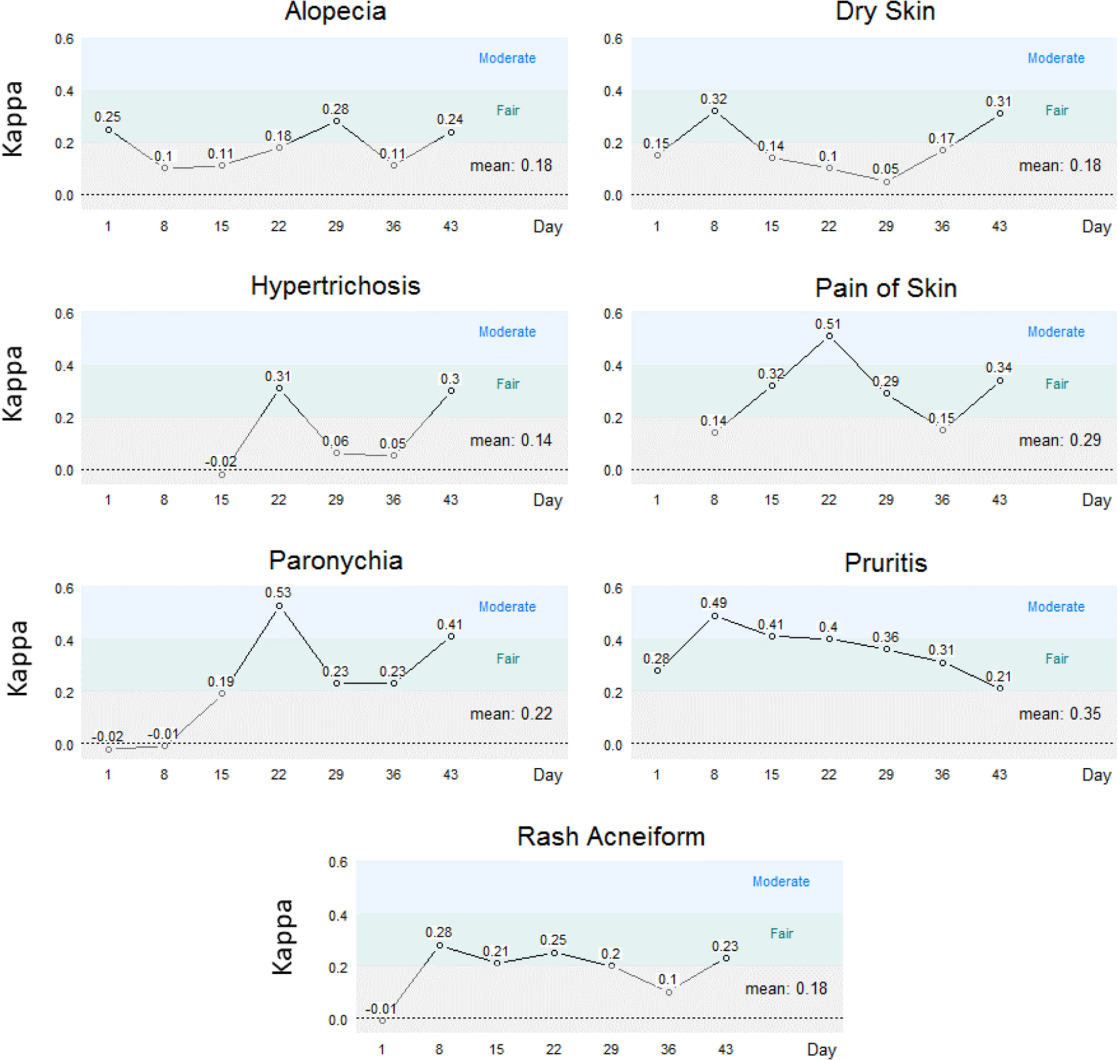
Criterion Validity For Each Symptom Based on Kappa Statistics Overall (Mean) and by Assessment Day. Criterion validity for the seven treatment toxicity categories most commonly associated with EGFRI toxicity. Criterion validity is based on agreement between the FACT-EGFRI 18 and the CTCAE scoring systems, and was assessed both overall and by assessment day. Moderate or better agreement is defined as Kappa coefficients of ≥0.41

Kappa statistics ranged from − 0.02 to 0.53, varying by assessment time and skin symptom (Fig. 2). Overall, 26 of the 46 (57%) individual assessment-specific Kappa statistics were > 0.20, indicating fair agreement between FACT-EGFRI 18 scores and CTCAE scores. Moderate agreement (≥0.41) for selected timepoints was found for pain of skin, paronychia, and pruritus. Mean toxicity-specific Kappa scores exceeded 0.2 for pain of skin, paronychia, and pruritus only.

### Known groups validity

**Fig. 3:**
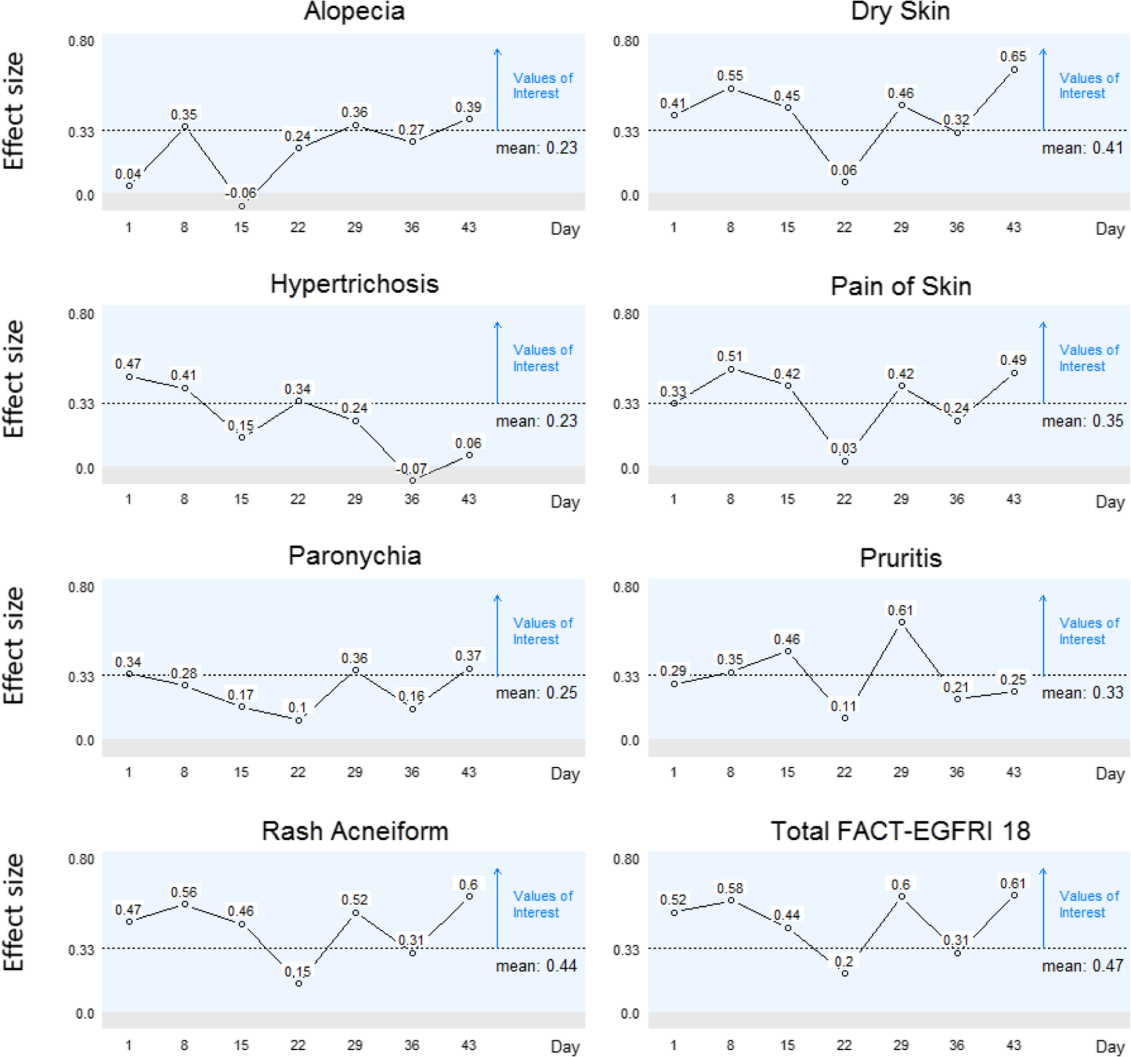
Standardized Values for Known Groups Validity. Standardized values for known groups validity. Known groups validity was assessed by examining differences in mean EGFRI subscores between patients who reported no symptoms (performance status = 0, *n* = 31) and any symptoms (performance status > 0, n = 93). ANOVA was used to compare FACT-EGFRI 18 scores between groups, with differences of 1/3 to 1/2 of a standard deviation considered of interest

Results of the ANOVA analysis are summarized in Fig. 3. Since higher FACT-EGFRI 18 subscores indicate better HRQL, positive standardized values (effect sizes) 0.33 or higher (MID) were considered of interest. Overall, 32 of the 56 (57%) individual assessment-specific results were > 0.33. Mean subscale scores were at least 0.33 for dry skin, pain of skin, pruritus, and rash acneiform, as well as for the total FACT-EGFRI 18 score. The full FACT-EGFRI 18 item scale was used to assess known groups validity, responsiveness to expected change, and reliability.

### Internal consistency reliability

**Fig. 4:**
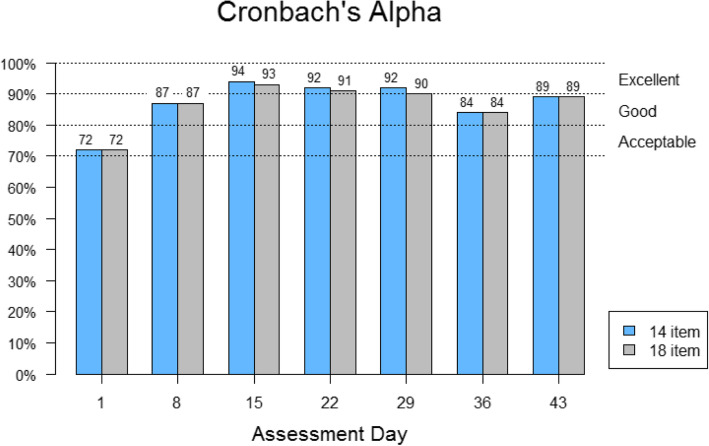
Cronbach’s alpha. Cronbach’s alpha is a measure of internal consistency reliability. Reliability was assessed for the FACT-EGFRI 14 14-item score for the 14 skin toxicities and the full FACT-EGFRI 18 scale of 18 items. Coefficients of > 0.70 are considered sufficient evidence of reliability

Cronbach’s alpha statistics are summarized in Fig. 4 by score and by time point. Reliability estimates were uniformly high (> 0.70) for both the FACT-EGFRI 14 and FACT-EGFRI 18, indicating strong internal consistency reliability, with every assessment time showing a Cronbach’s alpha > 0.70 for each scale.

### Responsiveness to change

**Fig. 5:**
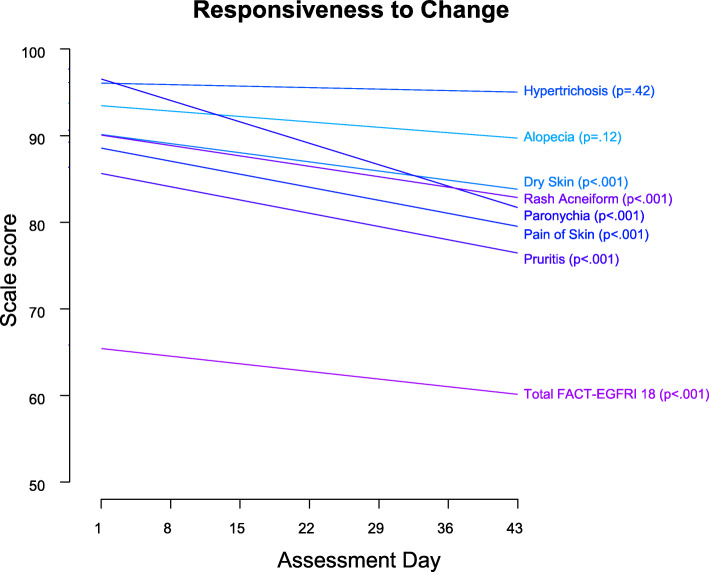
Responsiveness to change between Days 1 and 43

Figure 5 shows the linear mixed model fitted regression line results for responsiveness to change. The FACT-EGFRI 18 total score decreased significantly over time, consistent with a decrease in HRQL over time. Subscale scores decreased significantly (*p* < 0.001) over time for dry skin, rash acneiform, paronychia, pain of skin, and pruritus, but not for hypertrichosis (*p* = 0.42) or alopecia (*p* = 0.12).

### Minimal important difference (MID)

The mean difference between levels was 3.18 points for change in severity of skin symptoms, and 3.37 points for change in impact of skin symptoms (Table 3). Therefore, a reasonable estimate of the MID in the FACT-EGFRI 18 scores is 3 points. This estimate can be used to indicate the MID in future studies that include the FACT-EGFRI 18 measure [28].

**Table 3:** Mean Change in FACT-EGFRI by Patient-Reported Change in Severity and Impact of Skin Symptoms

	FACT EGFRI Total Score										
Assessment time	A lot better		A little bit better		About the same		A little bit worse		A lot worse		Mean Diff-erence^a^
	Mean	N	Mean	N	Mean	N	Mean	N	Mean	N	
**Change in SEVERITY of skin symptoms**											
Day 8			−0.50	4	0.86	69	−6.36	33	−17.53	11	
Day 15	4.50	2	−1.71	12	0.15	45	−6.96	47	−14.56	16	
Day 22	4.43	14	3.98	18	− 0.18	50	−3.67	36	−1.33	3	
Day 29	5.76	20	3.00	23	0.21	55	−2.03	21	−2.5	2	
Day 36	4.01	16	2.49	28	−0.31	48	0.53	22	−8	1	
Day 43	2.20	17	0.64	19	0.04	54	−4.52	21	−7.25	4	
Mean	4.17		1.32		0.13		−3.84		−8.53		3.18
**Change in IMPACT of skin symptoms on daily life**											
Day 8			−1.33	3	0.05	84	−9.17	23	−20.5	6	
Day 15	4.5	2	−0.4	10	−1.8	66	−8.73	36	−17.13	8	
Day 22	3.36	14	8.44	9	−0.75	79	−4.91	17	4	2	
Day 29	7.43	14	2.86	21	0.44	70	−2.57	14	−5	2	
Day 36	4.22	16	2.19	18	0.42	70	−1.2	11			
Day 43	1.91	18	1.23	13	−0.7	71	−5.1	10	−7.33	3	
mean	4.28		2.17		−0.39		−5.28		−9.19		3.37

^a^Unweighted mean difference calculated as the average of the difference between adjacent levels of patient-reported change categories. For instance, for severity, the difference is calculated as: mean of [4.17–1.32,1.32–0.13, 0.13-(−3.84), −3.84-(−8.53)]

### Association with treatment profiles

Patients who experienced a disruption in EGFRI treatment had lower FACT-EGFRI 18 scores than patients who did not have a disruption in EGFRI treatment by the end of the baseline period at assessment Day 22 (57.8 vs.62.2) and the end of the follow-up period at Day 43 (57.2 vs. 63.9).

## Discussion

When evaluating treatment regimens such as EGFRI that cause considerable skin toxicity that can negatively impact HRQL, it is important to have a validated instrument to assess the patient perspective. Our study showed uniformly high Cronbach’s alpha (> 0.70) for both FACT-EGFRI 14 (skin symptoms only) and FACT-EGFRI 18 across assessment times, indicating strong evidence of internal consistency among the items that make up these two composite scores. The results also demonstrated responsiveness to symptom change, and were correlated with treatment profiles. The known groups validity results were of interest for dry skin, pain of skin, pruritus, rash, and the total FACT-EGFRI 18 score. Although agreement (i.e. criterion validity) between individual and summary scales of the FACT-EGFRI 18 and the current clinician measure for assessing EGFRI skin toxicity (physician-rated CTCAE items) was generally only fair, this may reflect the limitations of the physician-reported CTCAE measure itself. The MID for the FACT-EGFR 18 was determined to be 3 points. The 2 items that reached the moderate agreement (≥0.41) for the criterion validity analysis were pain and pruritus, which are non-measurable and non-observable symptoms. Therefore, the inclusion of these 2 important skin-related symptoms in a PRO measure will be informative.

As previously noted, there are no currently available standardized PRO measures to quantify EGFRI-induced dermatologic adverse events and impact on functional status, from the patient’s perspective. Furthermore, the subjective nature of some of these EGFRI-induced papulopustular rash symptoms (i.e. pain of skin and itching) make it more appropriate and accurate to be assessed using the PRO approach. Dermatology-specific PROs such as the Skindex-29 [37] and the Dermatology Life Quality Index [38] are available and widely used, but these tools are not specifically designed to assess EGFRI-associated toxicity, such as hair change and paronychia. Therefore our results provide important data to support using the FACT-EGFRI 18 in EGFRI-treated patients to assess the impact of this specific class of treatment on HRQL.

This validation study included patients from across multiple oncology practice sites throughout the United States treated with a number of different EGFRI-containing treatment regimens. The included population was consistent with published experience with these agents, including incidence and time to development of the papulopustular rash [2–4]. However it included fewer than 20% non-white patients and was limited to English speaking patients, which somewhat limits generalizability. Overall these data are applicable to most community care settings and will be important for future clincial trials that include assessment with the FACT-EGFRI 18.

The FACT-EGFRI 18 provides data from the patient’s perspective yielding unique information as well as complementing clinician-rated CTCAE grades, especially for the symptoms of pain, pruritus, and paronychia. With the introduction of the NCI PRO-CTCAE since the initiation of this trial, additional research should be considered to optimize the utility of NCI PRO-CTCAE items in combination with the FACT-EGFRI 18 to best evaluate the EGFRI-induced papulopustular rash.

In conclusion, the psychometric results for the FACT-EGFRI 18 obtained in S1013 are supportive of use of this measure in English-speaking patients with cancer undergoing treatment with EGFRI-containing treatment regimens.

## Supplementary information

**Additional file 1.**

## Data Availability

The datasets generated and analyzed during the current study are not publicly available to help maintain confidentiality but are available from the SWOG data repository located at the SWOG Statistical Center in Seattle, WI.

## Abbreviations

ACRIN: American College of Radiology Imaging Network
CTCAE: Common Toxicity Criteria and Adverse Events
ECOG: Eastern Cooperative Oncology Group
EGFR: Epidermal Growth Factor Receptor
EGFRI: Epidermal Growth Factor Receptor Inhibitor
FACT: Functional Assessment of Cancer Therapy
FACIT: Functional Assessment of Chronic Illness Therapy
HRQL: Health-related Quality of Life
IADL: Instrumental Activities of Daily Living
MID: Minimal Important Difference
PRO: Patient Reported Outcome
PS: Performance Status
TKI: Tyrosine Kinase Small Molecule Inhibitor
