# Supplementary material for: A prospective study to validate the functional assessment of cancer therapy (FACT) for epidermal growth factor receptor inhibitor (EGFRI)-induced dermatologic toxicities FACT-EGFRI 18 questionnaire: SWOG S1013
Source: J Patient Rep Outcomes. 2020 Jul 8;4:54. doi: 10.1186/s41687-020-00220-x (PMC7343679; doi:10.1186/s41687-020-00220-x)
Supplement: Supplementary file 1 — Additional file 1. [file 41687_2020_220_MOESM1_ESM.docx]

**Online Appendix Table 1: Assessment Using Kappa Statistic**

|  | CTCAE version 4 grades | FACT-EGFRI items* | Outcome Measure Content |
| --- | --- | --- | --- |
| Rash acneiform | Grade 0-5 | ST4, ST5, ST6, ST9, ST17, ST22, ST24, ST26, ST32, ST34, ST37, ST38 | Total score, and each item individually |
| Pruritus | Grade 0-3 | ST6 and ST32 | Total score, and each item individually |
| Dry skin | Grade 0-3 | ST5, ST17, ST24, and ST37 | Total score, and each item individually |
| Pain of skin | Grade 0-3 | ST4, ST22, and ST32 | Total score, and each item individually |
| Paronychia | Grade 0-3 | ST15 and ST29 | Total score, and each item individually |
| Alopecia | Grade 0-2 | B5 | Individually |
| Hypertrichosis | Grade 0-2 | ST11 | Individually |
